# Evaluation of Convalescent Plasma in the Management of Critically Ill COVID-19 Patients (with No Detectable Neutralizing Antibodies Nab) in Kashmir, India

**DOI:** 10.3390/healthcare11030317

**Published:** 2023-01-20

**Authors:** Ahmed M. E. Elkhalifa, Showkat Ul Nabi, Naveed Nazir Shah, Khurshid Ahmad Dar, Syed Quibtiya, Showkeen Muzamil Bashir, Sofi Imtiyaz Ali, Syed Taifa, Iqra Hussain

**Affiliations:** 1 Department of Public Health, College of Health Sciences, Saudi Electronic University, Riyadh 11673, Saudi Arabia; 2 Department of Haematology, Faculty of Medical Laboratory Sciences, University of El Imam El Mahdi, Kosti 1158, Sudan; 3 Large Animal Diagnostic Laboratory, Department of Clinical Veterinary Medicine, Ethics & Jurisprudence, Faculty of Veterinary Sciences (FVSc) and Animal Husbandry (AH), Sher-e-Kashmir University of Agricultur-al Sciences and Technology of Kashmir (SKUAST-K), Shuhama, Alusteng, Srinagar 190006, Jammu & Kash-mir, India; 4 Department of Chest Medicine, Govt. Medical College, Srinagar 191202, Jammu & Kashmir, India; 5 Department of General Surgery, Sher-I-Kashmir Institute of Medical Sciences, Srinagar 190011, Jammu & Kashmir, India; 6 Biochemistry & Molecular Biology Lab, Division of Veterinary Biochemistry, Faculty of Veterinary Sciences (FVSc) and Animal Husbandry (AH), SKUAST-K, Shuhama, Alusteng, Srinagar 190006, Jammu & Kashmir, India; 7 Foot and Mouth Laboratory, Department of Clinical Veterinary Medicine, Ethics & Jurisprudence, Faculty of Veterinary Sciences (FVSc) and Animal Husbandry (AH), SKUAST-K, Shuhama, Alusteng, Srinagar 190006, Jammu & Kashmir, India

**Keywords:** convalescent plasma, COVID-19, best standard treatment, historical control, all-cause mortality, oxygen saturation

## Abstract

Background: For centuries, convalescent plasma (CP) has been recommended to treat a diverse set of viral diseases. Therefore, the present study was undertaken to evaluate the effectiveness of CP in critically ill COVID-19 patients. Methods and Materials: From 23 March 2021 to 29 December 2021, an open-label, prospective cohort, single-centre study was conducted at Chest Disease Hospital, Jammu and Kashmir, Srinagar. Patients with severe manifestation of coronavirus disease 2019 (COVID-19) under BST (best standard treatment) +CP were prospectively observed in order to evaluate effectiveness of CP therapy and historical control under BST were used as the control group Results: A total of 1667 patients were found positive for COVID-19. Of these, 873 (52.4%), 431 (28.8%), and 363 (21.8%) were moderately, severely, and critically ill, respectively. On 35th day post-infusion of CP, all-cause mortality was higher in the BST (best standard treatment) +CP group 12 (37.5%) compared to 127 (35%) in the BST group with an odds ratio (OR) of 1.4 and hazard ratio (HR) (95% CI: 1.08–1.79, *p* = 0.06). Similarly, 7 (21.9) patients in the BST+CP group and 121 (33.3) patients in the BST group showed the transition from critically ill to moderate disease with subhazard ratio (s-HR 1.37) (95% CI: 1.03–2.9). Conclusions: In the present study, we could not find any significant difference in the CP group and BST +CP in primary outcome of reducing all-cause mortality in critically ill patients with negligible Nabs levels. However, beneficial results were observed with use of CP in a limited number of secondary outcomes which includes days of hospitalization, negative conversion of SARS-CoV-2 on basis of RT-PCR on 7th day and 14th day, need for invasive mechanical ventilation on 14th day post-CP treatment, and resolution of shortness of breath.

## 1. Introduction

Since December 2019, SARS-CoV-2 (severe acute respiratory syndrome coronavirus 2) has caused the coronavirus disease 2019 (COVID-19) epidemic, which has engulfed the length and breadth of the world, resulting in widespread mortality [1,2,3]. The disease originated from Wuhan, China, and within three months, diseases were reported from almost every country [4,5,6,7]. The disease is manifested in mild, moderate, and severe forms with occasional involvement of multiple organ failure and death [8]. Throughout the world, healthcare workers and researchers are struggling to develop an effective therapeutic protocol to treat and contain the spread of COVID-19. In 2020, a meta-analysis was conducted and as per their study, CP has been found to be ineffective in non-COVID-19 severe respiratory viral infections which indicate indirect evidence of ineffectiveness of CP against COVID-19. These studies found that pooled estimate of four RCTs using CP against influenza have no significant improvement in ameliorating all-cause mortality [9]. Although several anti-viral and anti-protozoal drugs seem to be of clinical benefit in COVID-19, their efficacy is far from satisfactory [1]. To find the effective therapy against COVID-19, almost more than 300 drugs are being investigated under diverse clinical trials in different parts of the world. Still, treatments for COVID-19 are somewhat limited, and at present, the disease is managed mainly by supportive therapeutic strategies which comprises repurposing of anti-viral drugs, use of steroids, immune modulators, and drugs such as ivermectin [10]. Although some vaccine trials have observed promising results, these studies have postulated that these candidate vaccines can effectively protect against severe forms of COVID-19 [11]. However, SARS-CoV-2 virus has been found to mutate rapidly; henceforth, search for an effective vaccine remains an unending effort. Various drugs such as PAXLOVID (nirmatrelvir+ritonavir) have been approved against COVID-19; however, life threatening drug interactions have been reported in patients treated with PAXLOVID (). These studies indicate that despite the existence of anti-virals and other drugs found in the market and proposed to have ameliorative action against COVID-19, the understanding and use of antibodies (CP) could be a more effective solution in cases where the patient does not have time for the anti-virals to take effect.

Historically, convalescent plasma (CP) therapy has been used against poliomyelitis, measles, mumps, Ebola, SARS, and influenza with varying degrees of success [6,12]. Several countries have approved use of CP against COVID-19. For instance, the WHO has formulated guidelines against COVID-19 [13] and proposed use of CP as a potential therapeutic regimen against COVID-19. Similarly, countries such as the USA, Italy, Israel, and Spain have used CP in COVID-19 patients [14]. Subsequently, there are many reports of clinical benefits offered by CP therapy against COVID-19, but most of these clinical series have used CP in mild to moderate clinical presentations of COVID-19 [6,7,9,15]. At present, there are minimal numbers of published reports of CP used against critically ill COVID-19 patients; furthermore, these limited published reports have raised uncertainties regarding the safety and efficacy of CP as therapeutic options for critically sick COVID-19 patients. Although with the introduction of the vaccine, the number of deaths related to COVID-19 has shown some decline. Still, patients who have the critically severe manifestation of COVID-19 pose challenges to clinicians for their management and survival. Henceforth, based on the previous literature, the effectiveness of CP therapy should be evaluated in different subpopulations of COVID-19; hence, the present study was conducted to evaluate the effectiveness of CP therapy in critically ill COVID-19 subpopulation with negligible Nabs. The present study will help clinicians in deciding the utility of therapeutic interventions of CP therapy in subpopulations of COVID-19 with Nabs.

## 2. Material and Methods 

The present study was conducted in two phases. In the 1st phase, we identified risk factors for severe disease progression and death during intensive care as the outcome of the disease. In the 2nd phase, we evaluated the role of convalescent plasma therapy in critically ill COVID-19 patients. We reviewed the master data file and medical records of patients to obtain patient information on the standardized data form. The variables considered in the present study included age, sex, symptoms specific for COVID-19, radiological observations as per the procedure of our earlier publications [2,16], anti-viral/symptomatic treatment used, comorbidities of COVID-19 patients, biochemical parameters, duration of symptoms, and duration of hospitalization. The age of the patients was found to vary between 52 and 88 (68 ± 22) years in the CP group and between 56 and 92 years (78 ± 16) in the BST group.

### 2.1. Definition of Critically Ill Patients

Critically ill COVID-19 patients were defined as patients who met ARDS (acute respiratory distress syndrome) diagnostic guidelines [3,17]. Briefly, patients who met any of the following criteria were classified as critical COVID-19 patients:(1) respiratory failure and requiring mechanical ventilation; (2) shock, identified by the use of vasopressor therapy and elevated lactate levels (>2 mmol/L) despite adequate fluid therapy; and(3) with other organ failures that required intensive care management of the disease.

### 2.2. Phase-I: Identification of Risk Factors in the Retrospective Historical Control

A single-centre study of confirmed COVID-19 patients admitted to Chest Disease Hospital, Jammu and Kashmir, Srinagar, was conducted from 23 March 2020 to 30 November 2021. Patients in retrospective historical control were divided as per the severity of disease as moderately, severely, and critically ill COVID-19 patients. These patients were used to identify risk factors for the severe outcome of COVID-19 disease. COVID-19 disease was classified as severe, moderate, and mild as per guidelines of the Chinese National Health Commission 2020 management guidelines for COVID-19 (version 6.0) [18] and WHO Interim Guidelines [19].

To identify biochemical parameters having individual or combined effects associated with severe outcome/mortality in COVID-19, receiving operating curve (ROC) with the area under the curve (AUC) of 95% CI was constructed. ROC was conducted with the need for mechanical ventilation designated as positive and weaning off ventilation as negative. Similarly, death was designated as positive and discharge as negative. To further validate the identification of risk factors, violin plots were constructed, and statistically significant trends in clinical-biochemical parameters were identified.

### 2.3. Detection of Anti-SARS-CoV-2 Neutralizing Antibodies

Specific anti-SARS-CoV-2 neutralizing antibodies were detected using a commercial ELISA kit (Elabscience). Briefly, the ELISA kit was used based on the detection of anti-SARS-CoV-2 neutralization antibodies and the test was conducted as per the guidelines of the instruction manual.

### 2.4. Phase II: Evaluation of CP in Severely Ill COVID-19 Patients

#### Best Standard Treatment

The BST comprised of supportive and symptomatic treatment based on national COVID-19 guidelines and established hospital practice. The main category of drugs included antibiotics/anti-virals/immunomodulators and other medications.

### 2.5. Calculation of Sample Size

In the present study, we did not perform prior sample size calculations. The availability of convalescent plasmarecipients (n = 35) was considered a convenient sample of patients treated with CP. The control sample size (1:10) was calculated from an online calculator available at [20]. We assumed superiority of plasma therapy compared to BST with the dichotomous outcome (death or discharge from hospital). The true mean cure rates of plasma therapy and BST considered in the present study were 71% (pT = 0.85) and 66% (pC = 0.65), respectively, where pT denotes proportions of the treatment group and pC denote the proportion of the control group [8]. For achieving 80% power (i.e., 1 – β = 0.8) and 5% (α = 0.05) level of significance with allocation of 1:10 in the plasma therapy and BST groups (k = 0.10), 1– β represents the power of the test and ranges between 0 and 1 and α represents the level of significance. The sample size for the BST group was 360 with a dropout rate of 1.73%. Furthermore, three patients from the plasma therapy group were shifted to the BST group due to withdrawal of consent to undergo CP therapy.

### 2.6. Initiation of CP Therapy

To evaluate the beneficial role of CP therapy, COVID-19 patients admitted after initiation of plasma therapy at Chest Disease Hospital were retrospectively and prospectively observed into two groups, viz., BST +CP (n = 32) group and BST (n = 363) group. The recipients and donors of CP therapy were screened for eligibility criterion. Only those patients from which informed consent was obtained were considered for CP therapy. They were distributed into the plasma therapy and BST groups by computer-generated numbers (1:10). The prospective CP therapy was performed via block randomization of 11 patients in each block. The ten patients from each block were retrospectively assigned to the BST group and one patient prospectively to the plasma therapy group. Both patients and clinicians were aware of the treatment assigned to each patient. Patients were made aware of therapeutic intervention, and accordingly, informed consent was obtained from patients or their near relatives. The study was approved by the institutional ethical committee (CDSCO U/P No: EC/NEW/INST/2020/7452/01)in compliance duly with the Declaration of Helsinki.

### 2.7. Donor

Convalescent plasma was procured after a median of 35 (IQR-25, 45) days from SARS-CoV-2 diagnosis in the donor population following a standard protocol approved by the FDA [21]. Convalescent plasma was collected from donors (Supplementary Table S1/Supplementary Section S1.2). Accordingly, in the present study, we selected only those donors (for CP donation) having higher levels of neutralizing antibodies (more than 1:640) on the day of donation of plasma. The donation populations were aged 45.4 ± 13.4 (14 males and 18 females). Furthermore, the inversion of the threshold dilution with 50% inhibition was considered as Nab titer and inhibition rate of 20% was considered positive for Nabs. Donors were invited for plasma donation and were aged 45.4 ± 13.4 (14 males and 18 females). A total of 200 mL convalescent plasma was collected from donors (Table S1) recovered from SARS-CoV-2 after obtaining written consent from them. The criteria were as per [22], which includes (i) patients recovered from COVID-19 and tested negative for SARS-CoV-2 by two consecutive RT-PCR, (ii) patients having SARS-CoV-2 specific neutralizing antibody titer >1:640, (iii) donors tested negative for major blood transmitting diseases which include HIV/AIDS, syphilis, hepatitis, and other viral/ bacterial pathogens transmitted through plasma, and (iv) patients with no history of fever from the last ten days preceding plasma donation. Plasma recovered from donors was transferred to recipients on the same day.

### 2.8. Recipients

For plasma therapy, critically ill COVID-19 patients were considered in the present study. For that purpose, patients were defined as infected with COVID-19 on quantitative reverse transcriptase-polymerase chain reaction (RT-PCR) for SARS-CoV-2 from a nasopharyngeal swab. The selection of patients’ population who will receive CP was based on critical manifestation of disease and presence of Nabs below threshold level of less than 1:40. The recipient population was aged between 68 ± 22 (52 and 88) and we attempted to infuse CP in aged/sex matched patients. One day before transfusion, neutralizing antibodies specific for SARS-CoV-2 was estimated in the recipient population, and those patients were included, which had titer of Nabs, below 1:40. Furthermore, blood typing for compatible transfusion was conducted for ABO blood groups. All recipient population was advised to continue anti-viral and other symptomatic drug preparations prescribed previously.

### 2.9. Plasma Infusion

After cross-matching of recipient RBCs with donor plasma for compatibility testing, a single transfusion of 200 mL CP was infused @ 10–20 mL/kg body weight. Transfusion was adjusted in patients based on patients’ risk of volume overload, and during transfusion, patients were closely monitored by a physician for any adverse reaction.

### 2.10. Clinical Outcome

A primary clinical outcome was defined as all-cause mortality during ICU hospitalization. Patients were discharged from the hospital based on negative results for SARS-CoV-2 by two consecutive RT-PCR tests conducted two days apart, no respiratory symptoms specific for COVID-19, and resolution of inflammation as per pulmonary CT scan improvement. The secondary outcome was defined as improved oxygen saturation and weaning off/significant reduction in the need for mechanical intubation. The short-term safety study was conducted based on the hematology, death, and discontinuation of treatment, liver, and kidney function test.

### 2.11. Statistical Analysis

Continuous variables were indicated as median with range, while categorical variables were indicated as counts/percentage, which were compared using Chi-square test. Statistical significance between different groups was calculated using Mann–Whitney U test and a difference was considered to be significant when the *p* value was less than 0.05. Subgroup analysis was performed by Chi-square and Kruskal–Wallis H test for categorical and ordinal data, respectively. In the present study, OR (odds ratio) and HR (hazard ratio) were presented with 95% C.I.s and Chi-square *p* values. Ordinal primary and secondary outcomes were evaluated using an ordinal logistic regression model to estimate the odds ratio between arms on the 35th day post-treatment. Deaths within 35 days were censored to consider death as a competing event. Cox regression model was used to evaluate time to death or clinical improvement to evaluate hazard ratios (HR). An exemplary and grey regression model was used to estimate subhazard ratios considering death as confounding for its association. The area under the curve (AUC) and the 95% CI of the receiver operator characteristic (ROC) curve were computed with recovery as negative and death as positive to detect which biochemical parameter/s are more sensitive in predicting the severity of illness. The optimal cut-off for biochemical parameters was used to predict the severity of disease determined by Youden’s index. Data were analyzed by SPSS and results were expressed as mean and standard error.

## 3. Results

### 3.1. Baseline Characteristics of Patients Treated with BST +CP vs. BST Treatment

From 23 March to 29 November 2020, a total of 1667 patients were found positive for COVID-19 at Chest Disease Hospital, Jammu and Kashmir, Srinagar, India. Of these, 873 (52.4%), 431 (28.8%), and 363 (21.8%) were moderately, severely, and critically ill, respectively. The criteria for classification of patient population into moderately, severely, and critically ill were conducted as per guidelines of the Chinese National Health Commission 2020 management guidelines for COVID-19 (version 6.0) [18] and WHO Interim Guidelines [19]. The most prominent clinical manifestations in patients with COVID-19 were cough, sore throat, fever, and rhinitis at the time of hospitalization. Patients who died during hospitalization and patients with the critically severe manifestation of COVID-19 illness were associated with a higher degree of comorbidities than moderately ill/severe patients. A BST group was selected from critically ill patients hospitalized in the same hospital. The patients in the BST group were matched by age, sex, and need for high flow ventilator support with the BST+CP patient group. Following this, 32 critically severe COVID-19 patients (22 males and 10 females) with a median age of 68.3 ± 22.4 (52–88) were included in a prospective cohort study to receive CP. The mean time from onset of symptoms to infusion of CP transfusion was 3.2 days. Patients were treated with CP within 3 days of onset of severe manifestation of the disease, as earlier studies have reported beneficial outcome of CP therapy if initiated within 3 days of severe manifestation of the disease [22,23]. At the time of initiation of CP, no significant difference was observed between baseline characteristics of the BST group and a prospective cohort of the CP group, except for a higher prevalence of hypertension and rhinitis in BST (Table 1, Table 2 and Table 3). In both the study groups, patients were managed similarly except for CP therapy in the BST+CP arm in addition to BST.

### 3.2. Identification of Risk Factors for the Severe Outcome of the Disease

**Figure 1:**
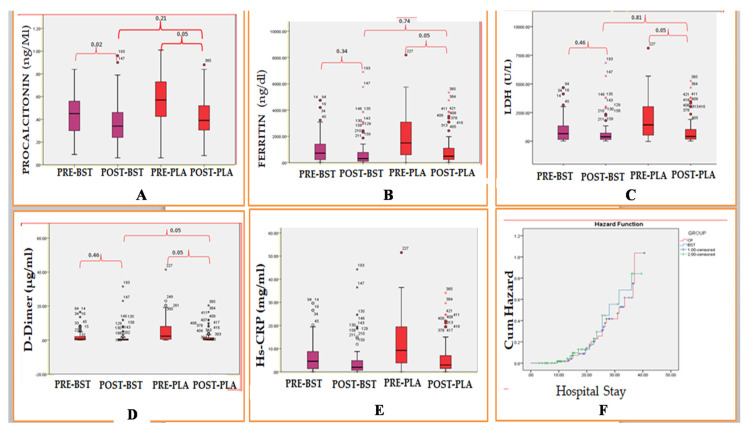
(**A**–**E**) Dynamic changes of Hs-CRP, D-dimer, LDH, ferritin, and procalcitonin during hospitalization and the horizontal lines represent the median value in each group. (**F**) Kaplan–Meier analysis showing hazard in CP and BST group.

In the present study, a statistically increasing trend was observed in high-sensitivity C–reactive protein (Hs-CRP), ferritin, and creatine phosphokinase (CPK), with the lowest level observed in moderate followed by severe and significantly highest levels in critically ill groups. Among biochemical parameters considered in the present study, D-dimer, Hs-CRP, ferritin, procalcitonin, and CPK were statistically significant among the three groups considered in the present study (Figure 1A–C). Furthermore, these findings are supported by ROC analysis, with AUC being highest for ferritin, Hs-CRP, and CPK (Figure 1D). Plasma D-dimer (y = 0.39x + 6.2; R^2^ = 0.63; *p* ≤ 0.05), Hs-CRP (y = 50x + 1.1; R^2^ = 0.79; *p* ≤ 0.01), ferritin (y = 0.006x + 14.8; R^2^ = 0.85; *p* ≤ 0.05), and LDH (y = 0.006x + 15.1; R^2^ = 0.82; *p* ≤ 0.01) levels in patients with COVID-19 correlated significantly with number of days of hospitalization (Figure 1E–H). Following this, the present study results indicate that D-dimer, Hs-CRP, ferritin, procalcitonin, and CPK were significantly elevated in severely ill patients and critically ill patients compared to moderately ill COVID-19 patients. Furthermore, the levels of D-dimer, Hs-CRP, ferritin, procalcitonin, and CPK showed significant gradation across patients with different degrees of severity of disease, with levels being lowest in the moderately ill patients compared to levels in severely ill and critically severe patients. Similarly, higher levels of D-dimer, Hs-CRP, ferritin, procalcitonin, and CPK were found in critically severe patients compared to severe patients. In the present study, D-dimer, Hs-CRP, ferritin, procalcitonin, and CPK, after adjustment for complication and other covariates, were identified as independent risk factors for mortality and severe progression of COVID-19 disease. Complete details on identifying risk factors for the extreme outcome of the disease is provided in Figure S1A–H and Table S2, available in the Supplementary Materials.

### 3.3. Effects of CP Transfusion/BSTon Risk Factors Identified

In the current study, risk factors identified were found to vary according to the severity of COVID-19. On the 7th day post-transfusion, a significant difference was observed in procalcitonin levels in both the BST +CP group (*p* = 0.05) and the BST group (*p* = 0.02) compared to levels on the day of initiation of CP with values still above reference range (Figure 1A). However, no significant difference was observed in post-treatment values between the BST +CP and BST group (*p* = 0.21). Similarly, ferritin levels were significantly reduced in the CP group (*p* = 0.05) post-treatment compared to ferritin levels on the day of initiation of CP. At the same time, a non-significant difference was observed in post-treatment levels in the BST group (*p* = 0.34), and a non-significant difference (*p* = 0.74) was observed between the CP group and BST group (Figure 1B) on the 7th day post-initiation of CP. In concurrence with these findings, LDH levels in the BST+CP group were significantly reduced non-significantly (*p* = 0.05) on the 7th day post-treatment compared to levels on the day of initiation of CP. In contrast, no significant difference (*p* = 0.46) was observed in the BST group post-treatment compared to levels on the day of initiation of CP. Similarly, we could not observe any significant difference in levels of LDH on the 7th day post-treatment between the BST and BST +CP group (*p* = 0.81) (Figure 1C).

On similar lines, post-treatment levels of D-dimer were significantly (*p* = 0.05) reduced in both BST +CP groups on the 7th day of treatment. Furthermore, a significant (*p* = 0.05) difference was observed in post-treatment values of D-dimer between the BST and BST +CP groups, while no significant difference was observed in post-treatment values of Hs-CRP and CPK between the BST and BST +CP groups on the 7th day of treatment and no significant difference was observed in Hs-CRP and CPK when pretreatment values were compared with post-treatment values (Figure 1D,E). Similarly, in concurrence with these results, on the 7th day post-CP treatment, there was no significant improvement in lung CT scan findings when BST and BST +CP values were compared (Table 4).

### 3.4. Effects of CP Transfusion vs. BST on the Primary Outcome of the Disease

At the end of the study, there was no significant difference observed in the primary clinical outcome of the two groups based on intention-to-treat analysis. Primary outcomes considered in the present research, viz., hospitalization time and need for high flow mechanical ventilation were almost similar in both groups. Negative conversion of SARS-CoV-2 based on RT-PCR on the 7th day was 52 (14.3) vs. 7 (21) s-HR 0.97 (1.5–1.8) *p* = 0.03, on the 15th day 78 (17.9) vs. 12 (37.5) s-HR 1.97 (1.9–5.5) and SP0_2_ (%) (with post-treatment comparison on the 5th day) were significantly higher in the BST +CP group compared to the BST group (Table 5). Similarly, regression to moderate diseases was substantially higher in the BST+CP group compared to the BST group 121 (33.3) vs. 7 (21.9) s-HR 1.4 (1.03–2.90) 0.04 (Table 5). In the BST+CP group, 16 patients were discharged on the 8–12th day and 14 patients were released on the 12–15th day based on absence of pyrexia for 3 days preceding discharge and 2 negative results of RT-PCR. In the BST group, meantime of discharge (deaths excluded) from the hospital was 27 (14–35) days, while in the BST +CP group, meantime for hospital stay was 25 (17–39) days s-HR 0.94 (0.86–1.79) *p* = 0.67 (Table 5). In present study, for calculation of days (hospitalization) needed for recovery in both treatment arms, we excluded deaths because their inclusion could make results biased.

### 3.5. Effects of CP Transfusion vs. BST on Secondary Outcome of the Disease

In the present study, COVID-19 patients treated with BST +CP (n = 32) were compared with those treated with BST (n = 363) in survival/hazard analysis. Our research reports no significant difference in clinical outcome/endpoints between BST+CP patients and BST patients with crude analysis hazard ratio-1.4 (1.1–1.8) *p* = 0.06 (Figure 1F). On the 7th-day post-infusion, in most of the parameters considered, no significant difference was observed in secondary outcomes in the CP group compared to secondary effects observed in the BST group. Most of the secondary outcomes considered remained comparable in patients during this time interval in both groups. The secondary consequences including several patients showing retrogression to moderate disease were significantly higher in the BST group than in the CP group. The resolution of shortness of breath on the 7th day and the need for invasive mechanical ventilation on the 14th day post-enrollment were significantly higher in the BST arm than the CP arm. In the present study, 17 patients out of 32 were observed to have reduced oxygen demand than the need for oxygen 1 day before CP infusion. Among these 17 patients,8 patients were weaned off from mechanical ventilation and 5 patients were shifted from high flow ventilation to low flow ventilation. In comparison, four patients were shifted from high flow oxygen demand to medium flow oxygen demand intermittently (Table 5).

### 3.6. Computer-Assisted Tomography (CT)

**Figure 2:**
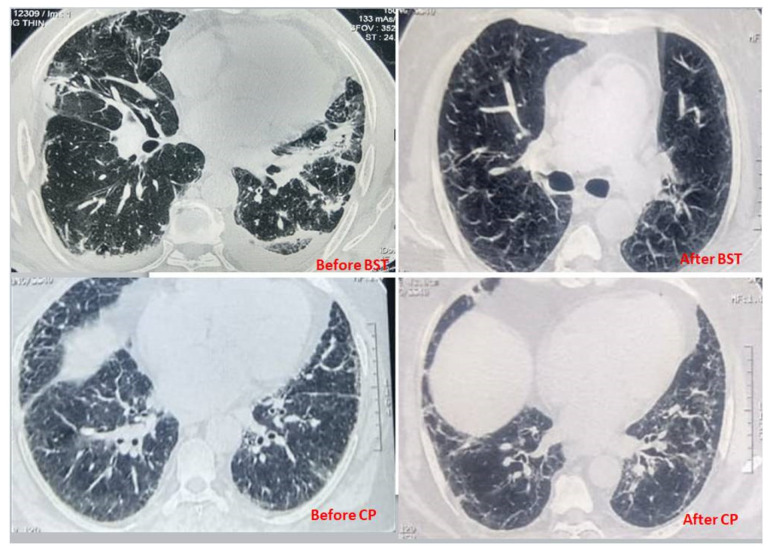
Chest CTs of patients before CP/BST therapy and after CP/BST therapy showing ground glass opacity, uneven density in multiple lung lobes, and multiple shadows of high density.

On CT scan, GGOs (ground glass opacities), pleural effusions, and pulmonary consolidations were significantly higher in critically ill patients than moderately ill patients. Patients in the BST group and CP group presented bilateral GGO (76% vs. 71%; *p* = 0.29), pleural effusion (34% vs. 27%; *p* = 0.23), and pulmonary consolidation (12% vs. 17%; *p* = 0.09) of relatively comparable degree (Figure 2). From these results, it can be seen that effects of CP transfusion on chest CT examinations observed on the 7th day post-infusion revealed 16 patients out of 32 patients showed no significant improvement in lung lesions compared to CT scan findings on the day of initiation of CP therapy. Similar results were observed in the BST group, in which 156 patients out of 363 showed no significant improvement in pulmonary lesions. When pulmonary lesion was compared between the CP and BST groups, patients in both groups had almost similar pulmonary lesions, except interstitial abnormalities (*p* = 0.03) and pulmonary consolidation (*p* = 0.02) which was significantly decreased in the CP group compared to the BST group. These findings are further supported by no significant increase in SPO_2_ levels (measured twice daily), an indirect marker for evaluating lung function improvement.

Adverse events reported in patients receiving CP were pain at the site of injection (2 patients), fever (5 patients), chills (3 patients), tachycardia (9 patients), bradycardia (12 patients), and laboured breathing (8 patients). Although deaths were observed in the CP group, we could not possibly attribute these deaths to CP transfusion.

## 4. Discussion

A wide range of drugs and vaccines are being evaluated against COVID-19, but in most cases, these claims remain to be evaluated by placebo-controlled double-blind clinical trials [6,8,24]. Various therapeutic and prophylactic strategies have been developed to contain the COVID-19 pandemic. Among them, vaccine development is high on the agenda despite the unknown duration of the protection time. Various vaccines have been used with promising results in different countries. The protective efficacy and the short-term and long-term side effects of the vaccines are of significant concern. To the best of our knowledge, no drug or therapeutic intervention has been approved until today showing anti-viral effects against SARS-CoV-2 in critically ill COVID-19 patients. In addition to this, there are minimal numbers of studies that have estimated levels of neutralizing viral antibodies (Nabs) in the donor/recipient population before their inclusion in the study [6,25]. A certain minimum level of Nabs may be required in the donor population because of 10-fold dilution of antibodies after administration in the recipient population. However, this minimum level of necessary Nabs in the donor population for anti-viral action is yet to be established. This study was designed to evaluate the efficacy of convalescent plasma in COVID-19 recipient patients with negligible Nabs (less than 1:40). Accordingly, in the present study, we selected only those donors (for CP donation) having higher levels of neutralizing antibodies (more than 1:640) on the day of donation of plasma. Our results indicate the beneficial role of CP in terms of resolution of SARS-CoV-2 infection based on RT-PCR on the 7th day and on the 14th day, and SP0_2_ (%), pro-inflammatory markers (ferritin, D-dimer, LDH), resolution of shortness of breath, and need for invasive mechanical ventilation on the 14th day post-enrollment. However, we could not find any significant role of plasma therapy in reducing all-cause mortality in the CP group compared to the BST group in critically ill patients having negligible Nabs levels. Furthermore, in the literature survey, drugs that were used in COVID-19 patients of the current study were found to have non-significant effect on modulation of the action of neutralizing antibodies [26].

In the present study, fever, cough, shortness of breath, and sore throat were the most commonly reported clinical sign in critically ill COVID-19 patients; these findings mirror the results of [27]. Primary outcomes that varied significantly between the BST and CP arms were recovery from SARS-CoV-2 infection based on RT-PCR on the 7th day 52 (14.32) vs. 7 (21) s-HR 0.97 (1.48–1.85) *p* = 0.03, on the 15th day 78 (17.90) vs. 12 (37.5) s-HR 1.97 (1.90–5.51), and SP0_2_ (%) (with post-treatment comparison on the 5th day) being significantly higher in the CP arm compared to the BST arm. Furthermore, we found no significant difference in mortality pattern among severely ill COVID-19 patients treated with CP+BST compared to those treated with BST alone in this study. Similarly, except retrogression to moderate disease, resolution of shortness of breath on the 7th day, and need for invasive mechanical ventilation on the 14thday post-enrollment, there was no difference in secondary outcome during hospitalization of severely ill COVID-19 patients treated with CP in combination with BST compared to those treated with BST alone. However, in COVID-19 patients treated with CP that survived, early clearance of virus was reported compared to patients treated without CP. These findings support the hypothesis that CP has a viral neutralizing effect [28,29]. The severity of COVID-19 is attributed to two overlapping mechanisms which include cellular damage by viral pathogen and hyper activation of immune response which manifest in classical symptoms of COVID-19 [30,31]. Nabs serve to accelerate viral clearance and inhibit entry of virus in target cells and hence helps in early viral clearance [32,33].

Contrary to this, we could not find any pronounced significant difference in risk factors (inflammatory markers) identified in the CP + BST group compared to the BST alone group on the 7th day post-initiation of CP therapy, which indirectly explain no immunomodulatory effect of CP. Henceforth, CP does not offer beneficial endpoint results despite early viral clearance by CP. Recently, a meta-analysis (Cochrane review) was conducted by including almost 20 studies to support our results. The meta-analytic study concluded no significant improvement in mortality and clinical improvement in COVID-19 patients treated with CP compared to those treated with standardized therapeutic regimens [34]. Our findings are in concurrence with clinical trials conducted in China on both moderately ill and severely ill COVID-19 patients. The study’s outcome improved clinical manifestation of diseases in rather ill COVID-19 patients, while no significant improvement was reported in severely ill COVID-19 patients [23]. Furthermore, a clinical trial (ConCOVID) conducted in the Netherlands on 86 patients was prematurely terminated due to no significant improvement in mortality, clinical presentation, and severe disease progression [35]. An observational study on the large sample size of COVID-19 patients proposed benefits of CP, if treated within three days of onset of symptoms. Still, the validity of their finding is questionable because of the absence of controlled comparison [22,36].

CP has been used since early times for different types of diseases, but its use has not been standardized concerning donor selection and the amount of Nabs in CP and recipient population [11,37]. Some investigators have attributed varied responses of CP therapy in different diseases and across other patients in the same illness to lack of standardization and procedure control [7,12]. Our study used CP with Nabs above threshold levels from the donor population and transfused it in the recipient population with negligible Nabs. Contradictory findings of the present study might be attributed to the use of CP under different clinical settings, variable patient population, variable donor population, and lack of standardization and procedure control. The potential strength of the current study includes the controlled design of the study with donor population having levels of Nabs above threshold levels and recipient population with negligible Nabs. Similarly, one of the significant takeaways from the present study includes the potential anti-viral effect of CP in severely ill COVID-19 patients across different age groups, as in the present study, we observed early resolution of SARS-CoV-2 infection based on RT-PCR on the 7th day and on the 14th day post-CP therapy and the data were found to vary significantly compared to the BST group.

We acknowledge some of the limitations of the current study. First, the number of patients in the CP group was small and there was no placebo control group. Second, patients were treated by different drugs, so there is possibility of additional contribution by these drugs in therapeutic improvement. Third, the actual level of Nabs in donors could not be calculated. So, owing to these limitations, there is need for a randomized, placebo-controlled clinical trial on a large sample size of patients.

## 5. Conclusions

In summary, our results indicate the beneficial role of CP in terms of resolution of SARS-CoV-2 infection based on RT-PCR on the 7th day and on the 14th day, SP0_2_ (%), pro-inflammatory markers (ferritin, D-dimer, and LDH), resolution of shortness of breath, and need for invasive mechanical ventilation on the 14th day post-enrollment. In the present study, we could not find any significant role of plasma therapy in reducing all-cause mortality in the CP group compared to the BST group in critically ill patients having negligible Nabs levels.

## Figures and Tables

**Table 1:** Demographic and clinical characteristics of critically ill COVID-19 patients of best standard treatment group (n = 363) and convalescent plasma treatment group (n = 32).

Characteristics	Best Standard Treatment (n = 363)	Convalescent Plasma Treatment (n = 32)	*p* Values
Age (Mean Years: IQR)	78 ± 16 (56–92)	68 ± 22 (52–88)	0.27
Male	270 (74)	21 (66)	0.21
Cough	200 (55)	17 (53)	0.12
Productive Sputum	75 (21)	7 (22)	0.78
Sore throat	345 (95)	30 (94)	0.81
Fever	250 (69)	4 (12)	0.85
Anorexia	65 (18)	3 (9)	0.42
Rhinitis	90 (24.79)	3 (9)	0.24
Insomnia	50 (14)	3 (9)	0.60
Hymoptypsis	20 (6)	3 (9)	0.72
Dysgusia	25 (7)	3 (9)	0.87
Nausia	30 (8)	3 (9)	0.39
Diarhoea	70 (19)	8 (25)	0.13
Myalgia	130 (36)	9 (28)	0.47
Fatigue	85 (23)	6 (19)	0.53
Headache	145 (40)	18 (56)	0.01
Oro-pharyngeal congestion	67 (18)	6 (19)	0.13
**Co morbidities**			
Chronic Obstructive Pulmonary Disease	65 (17.91)	6 (19)	0.51
Asthma	52 (14)	3 (9)	0.25
Diabetes Mellitus	39 (11)	3 (9)	0.41
Cardio-myopathy	52 (14)	14 (44)	0.24
Chronic Kidney Disease	65 (18)	4 (13)	0.18
Chronic Liver Disease	52 (14)	6 (19)	0.61
Cardio Vascular Disease	44 (13)	3 (9)	0.31
Thyroid disorder	39 (11)	2 (6)	0.52
Setriod use	52 (14)	4 (12)	0.25
Smoker	123 (34)	13 (37)	0.32
Anemia	78 (21)	11 (34)	0.09
**Therapy**			
Hydoxychloroquine and Azithromycin	64 (18)	6 (19)	0.06
Prednisolone	72 (20)	8 (25)	0.73
Dexamethasone	176 (48)	17 (53)	0.61
Remdesivir	116 (32)	15 (47)	0.65
Favipiravir	24 (7)	3 (8)	0.34

**Table 2:** Baseline biochemical characteristics of critically ill COVID-19 patients of best standard treatment group (n = 363) and convalescent plasma treatment group (n = 32).

Characteristics	Best Standard Treatment (n = 363)	Convalescent Plasma Treatment (n = 32)	*p* Values
Heart Rate (beats/m)	83 ± 2	85 ± 2	0.65
Respiration Rate (breaths/m)	20 ± 0.32	20 ± 0.62	0.91
Temperature (C)	39 ± 0.92	37 ± 0.18	0.02
SP0_2_ (%)	84 ± 0.70	79 ± 2	0.46
Hemoglobin (g/dL)	11 ± 0.23	12 ± 0.36	0.81
White Blood Cell Count (10^6^/L)	8258 ± 376	7631 ± 384	0.90
Lymphocyte (10^6^/L)	1670 ± 132	1659 ± 162	0.91
Platelet Count (10^9^/L)	158 ± 13	201 ± 16	0.06
Creatinine (mg/dL)	1.44 ± 0.10	1.19 ± 0.07	0.03
Blood Sugar (mg/dL)	114 ± 4.829	82 ± 13	0.72
Bilirubin (mg/dL)	1.2 ± 0.07	1 ± 0.10	0.13
SGOT (IU/L)	138 ± 13	165 ± 13	0.34
SGPT (IU/L)	145 ± 12	167 ± 4	0.15
ALP (IU/L)	131 ± 4	170 ± 28	0.80
Protein (mg/dL)	8.00 ± 0.07	7 ± 0.19	0.31

**Table 3:** Baseline radiological features of critically ill COVID-19 patients of best standard treatment group (n = 363) and convalescent plasma treatment group (n = 32).

CT Scan Findings, n (%)	BST Arm (n = 363)	CP Arm (n = 32)	*p* Value
Ground glass opacity	234 (64.46%)	18 (56.25%)	0.15
Local patchy shadows	145 (39.94%)	11 (34.37%)	0.35
B/L patchy shadows	108 (29.75%)	9 (28.12%)	0.89
Interstitial abnormalities	167 (46%)	12 (37%)	0.03
Pulmonary consolidation	109 (30%)	7 (21.8%)	0.09
Pleural effusion	214 (58.95)	17 (53.12)	0.45

**Table 4:** Comparison of “risk factors identified” in best standard treatment arm and convalescent plasma arm.

Characteristics	Pre BST	Post BST	*p* Value	Pre CP	Post CP	*p* Value	*p* Value *
Procalcitonin (ng/mL)	0.54 (0.59–1.05)	0.39 (0.26–1.77)	0.05	0.61 (0.21–1.08)	0.32 (0.45–1.56)	0.02	0.21
Ferritin (ng/L)	1389 (567–2789)	867 (278–1409)	0.01	1581.45 (810–2945)	708 (451–1832)	0.001	0.74
LDH (IU/L)	830 (451–1890)	710 (510–1945)	0.46	864 (612–1903)	512 (289–1367)	0.05	0.81
D-Dimer (µg/mL)	2.18 (0.93–6.78)	1.34 (0.67–4.50)	0.46	2.61 (1.51–3.67)	1.56 (0.89–2.67)	0.05	0.05
Hs-CRP (IU/L)	5.68 (2.56–8.12)	3.71 (1.03–5.23)	0.01	6.01 (1.34–7.45)	3.78 (0.91–5.56)	0.01	0.67
CPK (IU/L)	385 (145–738)	107.34 (23–178)	0.01	413 (241–610)	89.71 (45–212)	0.01	0.34

Data are presented as median (first and third quartile),* intergroup post treatment analyses.

**Table 5:** Comparison of primary and secondary outcome in best standard treatment arm and convalescent plasma arm.

Characteristics	Post BST	Post CP	Odds/Subhazard Ratio (95% CI)	*p* Value
**Primary outcomes**				
Need for high flow Mechanical Ventilation n (%)	260 (69.69)	22 (68.75)	OR 1.08 (0.78–1.45)	0.98
Hospitalization (Days) mean (range)	25 (17–39)	27 (14–35)	s-HR 0.94 (0.86–1.79)	0.67
SP0_2_ (%) (with post treatment comparison on 5th day) mean (range)	84.90 (78–94)	92.78 (85–97)	s-HR 1.67 (1.09–2.08)	0.05
Negative conversion of SARS-CoV-2 on basis of RT-PCR on 7th day n (%)	52 (14.3)	7 (21)	s-HR 0.97 (1.5–1.8)	0.03
Negative conversion of SARS-CoV-2 on basis of RT-PCR on 14th day n (%)	78 (17.9)	12 (37.5)	s-HR 1.97 (1.9–5.5)	0.01
**Secondary outcomes**				
Retrogression to moderate diseases n (%)	121 (33.3)	7 (21.9)	s-HR 1.4 (1.03–2.90)	0.04
All cause mortality at 35 days n (%)	127 (34.98)	12 (37.5)	1.4 (1.1–1.8)	0.06
Resolution of cough on 7th day; n (%).	230 (63.36%)	20 (62.5%)	OR 0.72 (0.48 to 1.36)	0.69
Resolution of fever on 7th day; n (%).	193 (53.16%)	16 (50%)	OR 1.34 (0.89 to 2.54)	0.56
Resolution of myalgia on 7th day; n (%).	167 (46.78%)	19 (59.13%)	OR 1.34 (0.79 to 2.61)	0.53
Resolution of sore throat on 7th day; n (%).	186 (51.12%)	21 (65.62%)	OR 0.92 (0.89 to 2.71)	0.17
Resolution of shortness of breath on 7th day; n (%).	123 (33.88%)	17 (53.12%)	OR 1.78 (0.93–2.90)	0.03
Days of respiratory support post enrollment. mean (range)	15 (7–23)	12 (7–28)	OR 0.89 (1.48–3.312)	0.29
Need for invasive mechanical ventilation on 14th day post enrollment; n (%).	145 (39.94%)	9 (30.12%)	OR 1.15 (0.67 to 1.96)	0.05

## Data Availability

The datasets generated during and/or analyzed during the current study are available from the corresponding author upon reasonable request.

## Supplementary Materials

The following supporting information can be downloaded at: https://www.mdpi.com/article/10.3390/healthcare11030317/s1, Figure S1. S1-SD: The violin plots of CPK (IU/L), Hs-CRP (IU/L) and ferritin (IU/L) levels in patients with COVID-19 in the critically severe group, severe group and moderate group in India. The red dot in the violin is the median value, and the blue rectangle is the percentile 25th and 75th. Figure S1D: ROC analysis of CPK (IU/L), Hs-CRP (IU/L) and ferritin (IU/L) for prediction of COVID-19 mortality. # Present *p* ≤ 0.01 and ## presents *p* *p* ≤ 0.001 compared to Values in moderate illness group. S1E-S1H: Correlation Between Plasma biomarkers (hs-CRP, D-Dimer, ferritin and LDH) with duration at hospitalization for samples collected on admission; Table S1: compatibility characteristics of donor population used for a donation of plasma for plasma therapy; Table S2: ROC characteristics of plasma inflammatory markers in COVID-19 patients to identify risk factors and early markers of COVID-19 disease.
