# Supplementary material for: Evaluation of Convalescent Plasma in the Management of Critically Ill COVID-19 Patients (with No Detectable Neutralizing Antibodies Nab) in Kashmir, India
Source: Healthcare (Basel). 2023 Jan 20;11(3):317. doi: 10.3390/healthcare11030317 (PMC9914564; doi:10.3390/healthcare11030317)
Supplement: Supplementary file 1 [file healthcare-11-00317-s001.zip › healthcare-2101509-supplementary.pdf]

healthcare

MDPI

Supplementary Appendix

Ineffectiveness of Convalescent plasma in the management of critically ill COVID-19 patients (with no detectable neutralizing antibodies Nab) in Kashmir, India

Donors were invited for plasma donation and were aged 45.4 ± 13.4 (14 males and 18 females). 200 ml Convalescent plasma was collected from donors (Table-S1) recovered from SARS COV-2 after obtaining written consent from them. The criteria were as per (Shen et al., 2020), which includes (i) patients recovered from COVID-19 and tested negative for SARS COV-2 by two consecutive RT-PCR, (ii) patients having SARS-COV-2 specific neutralizing antibody titer >1:640 (iii) Donors tested negative for major blood transmitting diseases which include HIV/AIDS, syphilis, hepatitis and other viral/ bacterial pathogens transmitted through plasma (iv) Patients with no history of fever from last ten days preceding plasma donation. Plasma recovered from donors was transferred to recipients on the same day.

Table S1. compatibility characteristics of donor population used for a donation of plasma for plasma therapy.

Donor blood group	Number of Donors	Volume `Collected
A-Positive	4	200 ml
A-Negative	4	200 ml
B-Negative	6	200 ml
B-Positive	7	200 ml
O-Negative	1	200 ml
O-Positive	3	200 ml
AB-Negative	4	200 ml
AB-Positive	3	200 ml

Identification of risk factors for the severe outcome of the disease

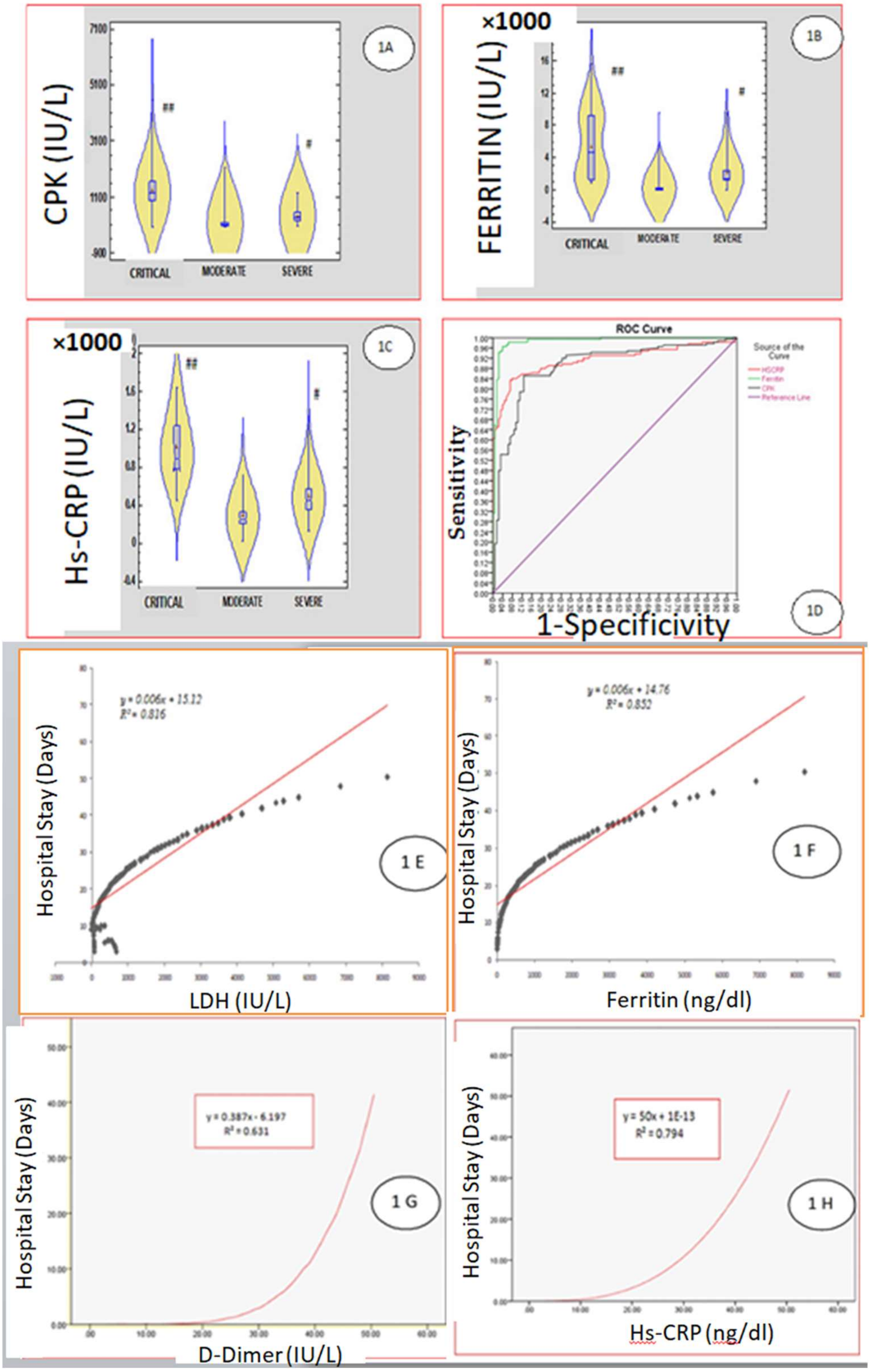

Figure S1. S1-SD : The violin plots of CPK (IU/L), Hs-CRP (IU/L) and ferritin (IU/L) levels in patients with COVID-19 in the critically severe group, severe group and moderate group in India. The red dot in the violin is the median value, and the blue rectangle is the percentile 25th and 75th. Figure S1D: ROC analysis of CPK (IU/L), Hs-CRP (IU/L) and ferritin (IU/L) for prediction of COVID-19 mortality. # Present p≤0.01 and ## presents p p≤0.001 compared to Values in moderate illness group. S1E-S1H: Correlation Between Plasma biomarkers (hs-CRP, D-Dimer, ferritin and LDH) with duration at hospitalization for samples collected on admission.

Table S2. ROC characteristics of plasma inflammatory markers in COVID-19 patients to identify risk factors and early markers of COVID-19 disease. .

Variable	AUC	95% CI	Cut-Off Value	Sensitivity	Specificity
Hs-CRP	0.91	0.89-0.94	14.4	0.85	0.89
Ferritin	0.98	0.97-0.99	478	0.96	0.96
CPK	0.89	0.85-0.93	226.5	0.84	0.88

References

1. Shen,C.;Wang,Z.;Zhao,F.;Yang,Y.;Li,J.;Yuan,J.;Wang,F.;Li,D.;Yang,M.;Xing,L.Treatmentof5criticallyillpatientswithCOVID-19withconvalescentplasma.*Jama* **2020**,*323*,1582–1589.
